# Effectiveness and safety of massage in the treatment of the congenital muscular torticollis: A systematic review and meta-analysis protocol

**DOI:** 10.1097/MD.0000000000021879

**Published:** 2020-08-28

**Authors:** Yuanyi Xiao, Zhenhai Chi, Fuqiang Yuan, Daocheng Zhu, Xilin Ouyang, Wei Xu, Jun Li, Zhaona Luo, Rixin Chen, Lin Jiao

**Affiliations:** a College of Acupuncture-Moxibustion and Tuina, Jiangxi University of Traditional Chinese Medicine; b The Affiliated Hospital of Jiangxi University of Traditional Chinese Medicine, Nanchang, China.

**Keywords:** congenital muscular torticollis, massage, protocol, systematic review

## Abstract

**Background:**

Massage has been widely used in the treatment of muscular torticollis in children, but there is no objective and systematic evaluation of the efficacy of various literature, and the efficacy of massage in the treatment of congenital muscular torticollis (CMT) is not clear. The purpose of this study is to evaluate the clinical efficacy and safety of massage in the treatment of muscular torticollis in children.

**Methods:**

Relevant randomized controlled trials (RCTs) will be searched from the databases of PubMed, the Cochrane Library, Embase, the China National Knowledge Infrastructure, Wanfang Database, Chinese Science and Technology Periodical Database, and Chinese Biomedical Literature Database from their inception to May 2020. Two reviewers will independently select studies, collect data, and assess the methodology quality by the Cochrane risk of bias tool. The RevMan V.5.3 will be used for meta-analysis.

**Results:**

This study will provide an assessment of the current state of Chinese massage therapy for the congenital muscular torticollis, aiming to show the efficacy and safety of massage treatment.

**Conclusion:**

This study will provide evidence to judge whether massage is an effective intervention for the third lumbar vertebrae transverse process syndrome.

**INPLASY registration number:**

INPLASY202070086.

## 1 Introduction

Congenital muscular torticollis (CMT), also known as synonymously with congenital torticollis, is a common disorder of the musculoskeletal system in neonates and infants.^[1]^ CMT is defined as torticollis caused by fibrosis contracture of the sternocleidomastoid muscle on one side. Its typical clinical characteristics are that the child's head is biased toward the affected side and the face is turned to the healthy side. CMT usually occurs during the neonatal period or after birth.^[2–4]^ Epidemiological studies have shown that the incidence of CMT is about 0.3% to 2%.^[5]^ In addition, recent studies have found that the incidence of CMT shows an upward trend.^[6]^ CMT is the third most common congenital musculoskeletal deformity in children after congenital hip dysplasia and calcaneovalgus feet, which has a serious impact on the quality of life of patients and their families.^[7,8]^ If CMT cannot be treated in time, the deformity will gradually become apparent as with age and the growth and development of skeletal muscle. In addition, secondary sequelae related to CMT including visual dysfunction, facial asymmetry,^[9]^ delayed development,^[10]^ cervical scoliosis, and vertebral wedge degeneration^[11]^ will have a serious impact on the child's appearance and even mental health.^[12]^

The actual etiology of CMT remains unknown, but evidence suggests it is closely related to positioning in utero, or trauma during delivery.^[13]^ Although several theories have been proposed to explain its occurrence, many controversies remain.^[14,11]^ At present, the treatment of CMT is mainly includes surgical treatment and conservative intervention. Surgical treatment is suitable for children who are not obvious for conservative treatment and older children.^[15]^ Although it can improve the clinical symptoms of CMT to a certain extent, surgical treatment has a high risk, is not easy to tolerate, and there are many complications, such as bleeding and nerve damage, infection, etc.^[16]^ Conservative treatment usually includes exercise therapy and family education5. Although there are studies showing that the above-mentioned therapies can improve the symptoms of CMT, there are few related research reports that need further study.^[17,18]^ Therefore, we need to find a more effective, more acceptable, and safe alternative treatment. Studies have shown that early intervention in physical therapy services for children younger than 1 month can achieve a 98% success rate by 2.5 months of age.^[12]^ As a common physical therapy method, massage manipulation is widely used in the treatment of CMT, and its clinical efficacy is positive.

Massage, systematically and systematically manipulate body tissues by hand to affect the nerves, muscle system, and circulation. It has been widely used in clinical treatment.^[19]^ At present, massage therapy is recognized as the preferred method for the treatment of CMT in my country, which can improve the cure rate of non-surgical treatment. Tuina therapy has unique advantages in clinical practice due to its features of green safety, precise curative effect, and high patient compliance.^[20,21]^ In 2018, the APTA Pediatric Physical Therapy Society (APPT) supported the update of CMT's clinical practice guidelines (CPGs), and PTS also recommended manual traction as the preferred treatment for CMT[2]. In addition, in many published clinical studies on massage treatment of children with CMT, most studies have shown that massage is a reliable method of treating CMT. Studies have shown that massage in the treatment of CMT raises the temperature of local tissues, dilates capillaries, accelerates the circulation of blood and lymph, promotes the absorption of local tissue metabolism and mass inflammation, improves the nutritional supply of surrounding muscle groups, promotes their growth and development, and relieves.^[20]^

The previous systematic review discussed related issues.^[21]^ However, the focus of this study is the efficacy of massage therapy for CMT-related clinical symptoms, and it does not focus on the effect of massage therapy on the efficacy of CMT imaging.^[22]^ Therefore, we conducted this study to systematically evaluate the impact of massage on CMT imaging. It can provide a basis for the diagnosis and treatment of massage therapy CMT.

## 2 Methods

### 2.1 Criteria for inclusion

#### 2.1.1 Type of studies

All the RCTs of Massage (Chinese Tuina) for the management of children with CMT patients will be included without publication status restriction or writing language. Semi-randomized controlled trials and animal studies, conference abstracts will be excluded.

#### 2.1.2 Type of participant

All cases included in the trial involved participants who had been diagnosed with CMT, diagnostic criteria for CMT, according to “Schools of pediatric massage,”^[23]^ “tuinaology,”^[24]^ and “the 2018 Congenital Muscular Torticollis Clinical Practice Guideline.”^[2]^ The diagnosis relies mainly on clinical and physical examination findings.^[25]^ It includes a persistent lateral flexion of the head to the affected side and cervical rotation to the opposite side; a palpable, intramuscular, fibrotic mass (fibromatosis colli) in the affected sternocleidomastoid (SCM) muscle; secondary sequelae include plagiocephaly, facial asymmetry, and developmental delay. The affected side is smaller than the healthy side; also, excludes the visual impairment compensated for postural torticollis, spinal deformity caused by bony torticollis, and nerve torticollis caused by cervical muscle paralysis. There is no restriction of age, sex, or race limit.

#### 2.1.3 Type of intervention

##### 2.1.3.1 Experimental interventions

The interventions of the experimental group will include any type of clinical massage for CMT alone to improve the symptoms. It includes Chinese massage, tuina, acupressure, therapeutic massage, general massage, acupressure, relaxation, etc. Massage combined with other interventions such as acupuncture, moxibustion, herbal medicine, qigong, functional exercise, and other mixed therapies will be excluded.

##### 2.1.3.2 Control interventions

The controlled intervention accepts any international recognized therapy, such as traditional medicine, acupuncture, etc. Non-intervention and Placebo will also be included. Studies comparing the therapeutic effects of different types of Massage manipulations will be excluded.

#### 2.1.4 Types of outcome measurements

##### 2.1.4.1 Primary outcome

Head posture range of motion (ROM) and neck rotation cervical ROM will be adopted as the primary outcomes. Changes of head deflection angle, active-passive cervical ROM between before treatment and follow-up, measured by the clinical examination. A study indicates that decisions guiding treatment for CMT are based on clinical examination rather than on ultrasound findings.^[26]^

##### 2.1.4.2 Secondary outcomes

1. Use ultrasound and color Doppler imaging to observe change of fibrotic mass in the affected SCM muscle before treatment and during follow-up, such as the thickness, heterogenous echogenicity, and asymmetry of the SCM muscle, mass size. Many studies show that ultrasonography is generally accepted as the primary evaluation tool for CMT, can objectively respond to treatment effects.^[27]^
2. Change in clinical symptom score from baseline to the last available follow-up. The score included lateral head-righting, sternocleidomastoid tumor thickness, early motor skills, development of symmetrical movement, craniofacial asymmetry changes, parental stress, compliance with home exercises, overall improvement, and other possible outcomes potentially reflecting changes in the subject's condition.
3. The incidence rate of adverse events.

#### 2.1.5 Exclusion criteria

1. The experimental group without massage (Tuina) will be excluded;
2. Reviews, animal experiments, case report, and non-randomized controlled trials will be excluded;
3. The study comparing different forms of Massage for CMT, such as Massage versus manipulation, will be excluded.
4. The literature information is unclear and the full-text cannot be obtained for the next analysis, will be excluded.

### 2.2 Search methods for identification of studies

#### 2.2.1 Electronic data sources

Search from the establishment of the database to May 1, 2020. We will search 4 English medical electronic databases including PubMed, EMBASE, Cochrane Central Register of Controlled Trials, Web of Science. Chinese literature will be searched through China's 4 major databases, including Chinese Biomedical Literature Database (CBM), Wanfang Database (WF), the Chongqing VIP (VIP) and Chinese National Knowledge Infrastructure (CNKI). The searching strategy of PubMed is presented in Table 1.

**Table 1:**
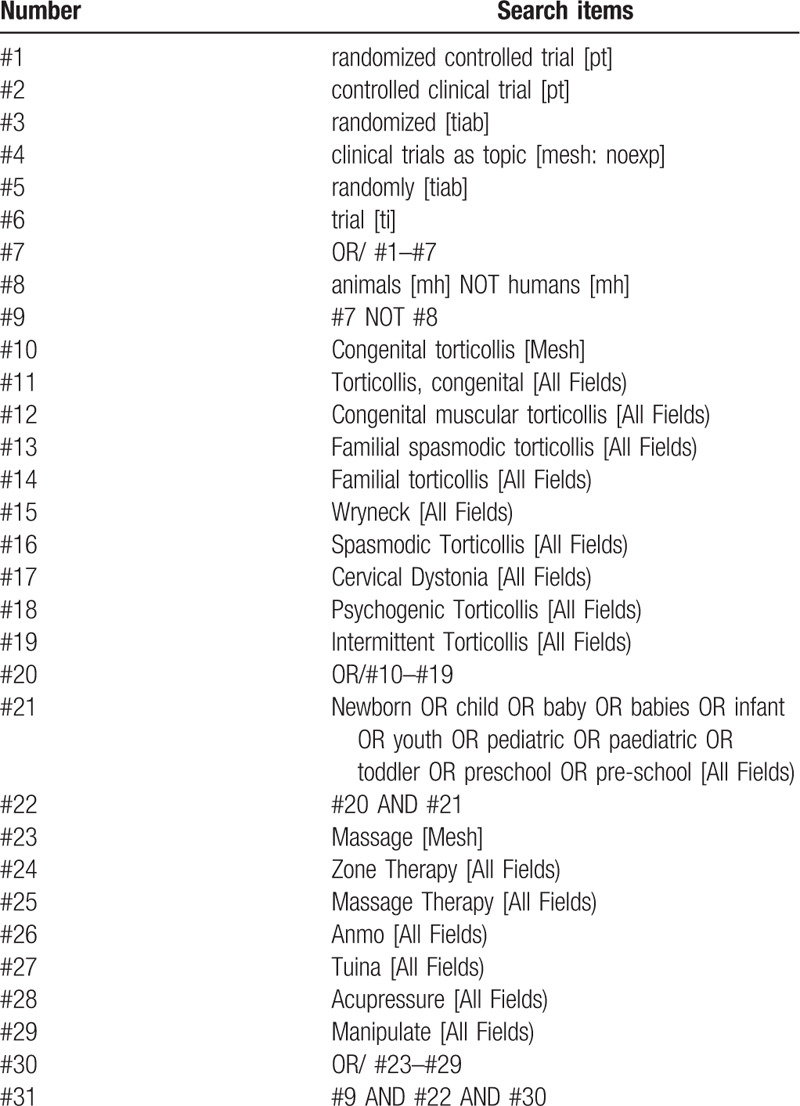
Search strategy used in PubMed database.

#### 2.2.2 Search other resources

We will search the Clinicaltrials.gov, China Clinical Trial Registry, and the relevant conference papers related to massage for treatment of congenital muscular torticollis, proposing to obtain unpublished, or ongoing has not uploaded trial data.

### 2.3 Data collection

#### 2.3.1 Selection of studies

**Figure 1:**
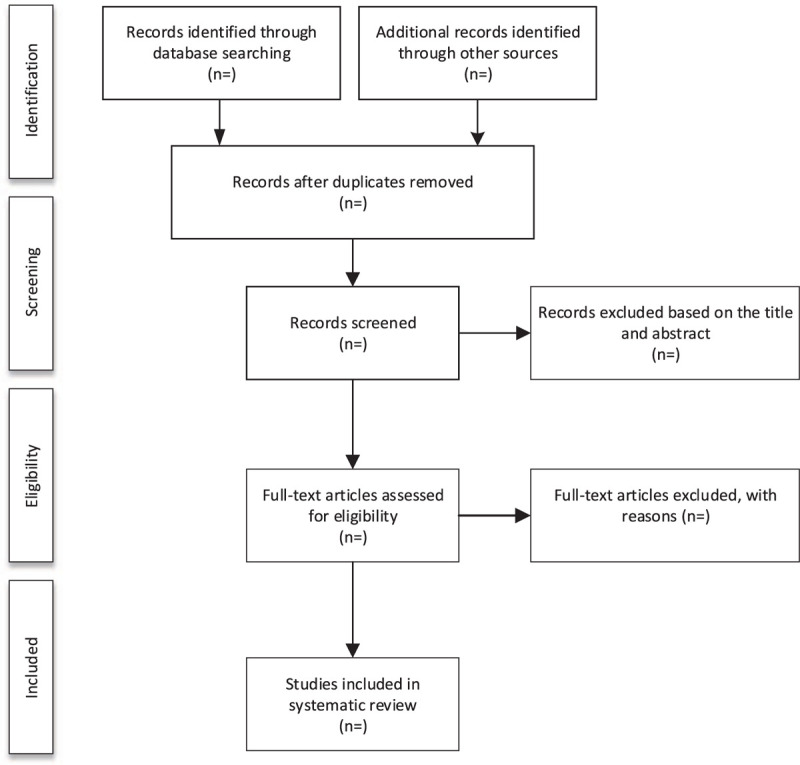
Flow diagram of study selection process.

We will export all the studies of electronic searches according to the search strategy into the EndNote software (V.X9) for management. Literatures obtained from other sources will also be imported into EndNote and remove the duplicate dates. Then, 2 researchers (YY and ZH) will independently screen the research literature that met the Eligibility criteria by reading the titles and abstracts. Irrelevant literature will be deleted. If they cannot determine whether can be included in the study by the title and abstract of the literature, the Full-text articles will be screened for judgment. After this step, the 2 researchers (YY and ZH) conducted a cross-check. During this process, if there appears any disagreement, it will be resolved through group discussions or decided by the third-party reviewer (FQ). The specific process and results of studies selection will be shown in the flow chart of Fig. 1.

#### 2.3.2 Data extraction and management

The research team will determine the standard data extraction form in advance, and then the 2 researchers (YY and ZH) will independently extract the following information from the included studies into the form:

1. Study basic information: Year of publication, Source and ID, the first author name, Publication language and Country, Article title of the study, etc.
2. Participant information: sex, age, country, ethnicity, diagnostic criteria, basic disease information, sample size, etc.
3. Trial characteristic: Number of groups, test design method, random control method, blind method, result analysis method, number of participants in observation and control groups, etc.
4. Interventions and Controls: The method of intervention, the name of the operation method, the duration and frequency of treatment, and the treatment cycle, name and type of the control, combination therapy, etc.
5. Outcomes: Various evaluation standards and types, primary and secondary outcomes (Change in Clinical symptom score, evaluation of imaging changes), timeline for assessment, length of follow- up period, adverse event, etc.

After extracting the data, the 2 researchers (YY and ZH) will conduct a cross-check. If there is any ambiguity, it will be discussed and decided by all members of the research group. The extracted data will be listed in the standard data table (Microsoft excel 2016), and FQ will check it to ensure the data are accurate.

### 2.4 Assessment of risk of bias in included studies

Two reviewers (FQ and DC) of the team will use the risk of bias assessment tool by the Cochrane Collaboration to independently evaluate the quality of the final included trials.^[28]^ The risk assessment indicators will include the following 7 contents: random sequence generation, allocation concealment, blinding of participants and personnel, blinding of outcome assessment, incomplete outcome data, selective reporting, and other sources of bias. Each domain of the study will be judged as high-risk, low-risk, and unclear risk of bias as the evaluation results. Our reviewers (FQ and DC) will strictly check the assessment results in accordance with the evaluation rules. If there is any ambiguity and disagreement, we will be resolved through consultation. In addition, it can also be judged by the third reviewer (XL).

### 2.5 Data synthesis

#### 2.5.1 Measures of treatment effect

We will designate 2 reviewers (YY and ZH) use Review Manager Software (RevMan 5.3) and Stata software to conduct statistical analysis and synthesize all data. For categorical data, we will use the risk ratio (RR) and 95% confidence intervals (CIs) to calculate and summarize data. For continuous data, mean difference (MD) and 95% CIs will be used to present the data synthesis outcome. If the outcome variables of different measurement scales are measured, standardized mean difference analysis (SMDS) with 95% CI will be performed.

#### 2.5.2 Management of missing data

If data are missing in the included study, we will contact the author by email or phone to obtain the original data and incomplete data. If the contact fails, we will follow the Cochrane Handbook methods and estimate the missing means and standard deviations of the baseline change based on existing baseline data and other data.^[29]^

#### 2.5.3 Assessment of heterogeneity

Chi-square test in forest plot and *I*^2^ statistic will be used to assess the heterogeneity. When performing a chi-square test, a *P* value <.10 will be considered significant.^[30]^ If performing *I*^2^ statistic verification, the effect of heterogeneity on the Meta-analysis will be quantified by calculating the *I*^2^ value. The specific *I*^2^ value follows the measurement standard as follows: if the *I*^2^ statistic is ≤50%, the research results might be considered no heterogeneity, and the fixed-effects model will be used for data synthesis and analysis; if the *I*^2^ statistic is 50% ≤ *I*^2^ ≤ 90%, means extensive heterogeneity; when 75% ≤ *I*^2^ ≤ 100% will be considered as important heterogeneity and a random effects model will be applied, and sensitivity analysis is used to assess the impact of all included trials on the final outcome results.

#### 2.5.4 Assessment of reporting biases

For the inclusion of >10 trials, a funnel plot will be drawn to detect the reporting bias. The publication bias for binary and the asymmetry of funnel plot will be assessed quantitatively using the Egger test.

#### 2.5.5 Subgroup analysis

If there are significant heterogeneities in the included studies, the STATA software will be used for subgroup analysis and meta-regression analysis according to the characteristics of the test subjects, sample size, different massage intervention methods, quality of included trials, etc.

#### 2.5.6 Sensitivity analysis

We will evaluate the robustness of the meta-analysis results through sensitivity analysis, and exclude such as small-sample trials and low-quality trials to explore the impact of trial quality on efficacy estimates. In addition, we will conduct a second meta-analysis based on the results of the sensitivity analysis, summarize in tables and discuss.

#### 2.5.7 Grading the quality of evidence

Two reviewers will independently evaluate the quality of the evidence of all research outcomes by using the Grading of Recommendations Assessment, Development and Evaluation (GRADE) system. And according to the GRADE rating standards, use “high,” “moderate,” “low,” “very low” 4 levels to rate the quality of evidence.^[31,32]^

#### 2.5.8 Ethics and dissemination

The second study based on the literature in this study does not involve the personal data of the research trials, so it does not require ethical approval. We will provide systematic evaluation and evidence by evaluating the treatment of TCM by Massage, and provide methods and ideas for the clinic. The systematic review and result will be published in a peer-reviewed journal.

## 3 Discussion

CMT is a common disease of infants and young children, mostly related to contracture or fibrosis of neck muscle and soft tissue. Massage is one of the widely accepted traditional Chinese medicine treatment techniques. Through the doctor's manipulation of the patient's neck muscle and soft tissue (including gentle, continuous, and vigorous application of force on the affected area), the purpose of relaxation is achieved. Studies have shown that massage can effectively relieve muscle cramps in CMT patients and increase their neck mobility. Massage has been widely used to treat CMT, is more acceptable without pain, and has higher compliance.^[33]^ More and more countries use massage as a supplementary option. In recent years, many related clinical studies have shown that massage can effectively relieve CMT symptoms, and can replace surgery as a first-line CMT treatment.^[6]^ In addition, the “Clinical Practice Guide” recommends TCM massage therapy as one of the preferred interventions for CMT.^[2]^ Therefore, the purpose of this study is to evaluate the clinical efficacy and safety of massage therapy for CMT. The conclusion of this study can provide evidence-based medical advice for massage therapy of CMT.

Limitations of research: first, during massage therapy, the choice of treatment, choice of treatment site, time and frequency of operation may be heterogeneous. Second, this study sets strict inclusion criteria, and the inclusion of high-quality literature may have less impact, the reliability of the systematic review largely depends on the comprehensiveness and methodological quality. Third, the included studies do not limit the language types, there are certain language biases.

## Author contributions

**Conceptualization:** Yuanyi Xiao, Zhenhai Chi.

**Data curation:** Yuanyi Xiao, Zhenhai Chi.

**Formal analysis:** Yuanyi Xiao, Fuqiang Yuan.

**Funding acquisition:** Yuanyi Xiao, Rixin Chen, Lin Jiao.

**Investigation:** Yuanyi Xiao, Zhenhai Chi.

**Methodology:** Yuanyi Xiao, Fuqiang Yuan.

**Project administration:** Lin Jiao.

**Software:** Yuanyi Xiao, Zhenhai Chi.

**Supervision:** Lin Jiao.

**Validation:** Lin Jiao.

**Visualization:** Yuanyi

**Writing – original draft:** Yuanyi Xiao, Fuqiang Yuan, Rixin Chen.

**Writing – review & editing:** Yuanyi Xiao, Zhenhai Chi, Fuqiang Yuan.
